# MRI-based assessment of the mylohyoid muscle in oral squamous cell carcinoma, a 7-point scoring method

**DOI:** 10.1007/s00330-024-11016-8

**Published:** 2024-08-29

**Authors:** E. Radin, A. V. Marcuzzo, J. de Groodt, F. Degrassi, L. Calderan, V. Ramella, G. Tirelli, M. Ukmar, M. A. Cova

**Affiliations:** 1 Department of Radiology, University of Trieste, Trieste, Italy; 2 ENT Clinic, Head and Neck Department, Azienda Sanitaria Universitaria Giuliano Isontina—ASUGI, Trieste, Italy; 3 Department of Radiology, Azienda Sanitaria Universitaria Giuliano Isontina—ASUGI, Trieste, Italy; 4 Department of Plastic, Reconstructive and Aesthetic Surgery, Azienda Sanitaria Universitaria Giuliano Isontina—ASUGI, University of Trieste, Trieste, Italy; 5 ENT Clinic, Head and Neck Department, Azienda Sanitaria Universitaria Giuliano Isontina—ASUGI, University of Trieste, Trieste, Italy; 6 Department of Radiology, Azienda Sanitaria Universitaria Giuliano Isontina—ASUGI, University of Trieste, Trieste, Italy

**Keywords:** Head and neck squamous cell carcinoma, Multiparametric MRI, Otolaryngology, Surgical oncology, Scoring methods

## Abstract

**Objectives:**

To investigate preoperative MRI evaluation of the features of the mylohyoid muscle (MM) predictive of its infiltration in oral squamous cell carcinoma (OSCC) treatment planning, defining the most appropriate sequences to study its deep extension into the floor of the mouth (FOM).

**Materials and methods:**

We applied a 7-point score to retrospectively evaluate preoperative imaging of patients who underwent surgery for OSCC over 11 years. The results were compared with histopathological findings using Spearman’s rank coefficient. Receiver operating characteristic curves were employed to assess the score’s ability to predict MM infiltration, determining optimal thresholds for sensitivity, specificity, and predictive values. The Mann–Whitney *U*-test confirmed that infiltration judgments did not overlap around this threshold. Cohen’s K statistical coefficient was used to evaluate the interobserver agreement.

**Results:**

Fifty-two patients (mean age 66.4 ± 11.9 years, 36 men) were evaluated. Histopathological examination found MM infiltration in 21% of cases (*n* = 11), with 90% classified in the highest Score categories. A score > 4 proved to be the best cut-off for predicting the risk of MM infiltration, with a sensitivity of 91% (CI: 0.57–0.99), specificity 61% (CI: 0.45–0.76), PPV 38% (CI: 0.21–0.59), and NPV 96% (CI: 0.78–0.99). At the subsequent single-sequence assessment, the TSE-T2wi had the highest diagnostic accuracy, with sensitivity 90% (CI: 0.57–0.99), specificity 70% (CI: 0.53–0.82), PPV 45% (CI: 0.25–0.67), and NPV 96% (CI: 0.80–0.99).

**Conclusion:**

The 7-point score is a promising predictor of safe surgical margins for MM in OSCC treatment, with the particular benefit of T2-weighted sequences.

**Clinical relevance statement:**

Our scoring system for tumor infiltration of MM, which is easy to use even for less experienced radiologists, allows for uniformity in radiological language, thereby ensuring crucial preoperative information for the surgeon.

**Key Points:**

- *The relationship of the MM to an oral lesion may impact surgical planning*.
- *As the score increases, there is a greater incidence of infiltration in the MM*.
- *Our score system improves radiologists’ reporting for MM involvement by tumor*.

## Introduction

The oral cavity represents a predominant site for head and neck malignancies, witnessing an annual incidence exceeding 350,000 cases globally, primarily characterized by oral cavity squamous cell carcinoma (OCSCC). This affliction notably affects males aged between 65 and 85, with the floor of the mouth (FOM) exhibiting heightened susceptibility in Western countries [1].

Recent updates to the TNM classification system (UICC/AJCC 8th edition) underscore the significance of the depth of invasion (DOI) in the stratification of oral cancer. A direct correlation exists between increased DOI and a heightened propensity for lymph node metastasis. This is particularly worrisome for advanced stages as it heightens the risk of local recurrence [2].

Magnetic resonance imaging (MRI) emerges as the preeminent modality for staging oral cavity squamous cell carcinoma (SCC), offering superior soft tissue contrast and lower susceptibility to dental artifacts in comparison to computed tomography (CT). This distinction is particularly noteworthy for tumors affecting the tongue and FOM [3, 4].

The mylohyoid muscle (MM) represents an anatomic and radiologic landmark of the oral cavity and suprahyoid region, supporting the FOM and separating the sublingual and submandibular spaces [5–7]. The anatomical proximity of lesions to the MM significantly influences the choice of surgical approach [5]. In instances where involvement is evident, an *en-bloc* resection coupled with reconstructive surgery utilizing a free flap aligns with prevailing standards of care [8].

Debate surrounds the comparative efficacy of *en-bloc* vs discontinuous resection methods. Historically, *en-bloc* resection has exhibited favorable prognostic outcomes [9, 10], albeit marked by its aggressive nature. Conversely, recent studies suggest promise in the less invasive approach of discontinuous resection, emphasizing its non-inferiority concerning local and locoregional control [8, 11–14].

Given the clinical and therapeutical value the MM may have in patients affected by oral squamous cell carcinoma (OSCC) of the FOM, our investigative pursuit involved the comprehensive analysis of preoperative MR images to detect morphological and signal alterations indicative of its infiltration. Subsequently, a 7-point scoring system was devised to establish a correlation between risk categories and histopathological reports, assessing diagnostic precision at both an initial multi-parametric and a subsequent sequence-selective evaluation.

## Materials and methods

### Population

This study was performed in line with the principles of the Declaration of Helsinki. Despite the retrospective nature of our work, the approval of our University’s local Ethics Committee was nevertheless sought and granted.

Between March 2010 and March 2021, 81 patients diagnosed with OSCC of the FOM underwent surgery at our Otorhinolaryngology and Head and Neck Surgery Department. Staging followed the current National Comprehensive Cancer Network—Head and Neck Cancers Guidelines [15].

For enrollment in our study, high-quality preoperative MRI and adequate histological reports for frozen sections or surgical specimens were necessary.

Consequently, 29 patients were excluded: 5 lacked preoperative exams, 19 had only preoperative CT available, 2 had missing information on MM in the histopathological reports, and 3 had severe imaging degradation due to artifacts.

### MR imaging and protocols

For all patients with a confirmed diagnosis of SCC with FOM involvement who had undergone surgery at the Otolaryngology Department of our hospital between March 2010 and March 2021, we re-evaluated the preoperative Head and Neck MRI acquired at the Diagnostic and Interventional Radiology Department.

Two radiologists with 2 years and 7 years of experience in Head and Neck imaging, blinded to the pathologic outcome, analyzed the preoperative images of the 52 patients in our sample. According to the scanner available, the head and neck MRI acquisition protocol in our department involves the following sequences acquired in three orthogonal planes:

- 3-T scanner (Ingenia, Philips Healthcare): TSE-Dixon-T2, TSE-T1, DWI (axial plane only), and fat-suppressed contrast-enhanced (FSCE) TSE-T1 (SPIR). Slice thickness 2.5 mm.
- 1.5-T scanner (Achieva, Philips Healthcare): TSE-T2, fat-suppressed (FS) TSE-T2 (STIR), TSE-T1, DWI (axial plane only), and FSCE TSE-T1 (SPIR). Slice thickness 3 mm.

Contrast-enhanced sequences were performed after intravenous administration of a macrocycle-structured gadolinium-based MRI contrast agent (DOTA-Gd).

### 7-Point-score

Parametric scoring systems are vital tools in clinical practice. Similar to the work of Kim et al on the pharyngeal constrictor muscle in oropharyngeal carcinoma [16], our proposed 7-point score could represent an easily reproducible tool to support radiologists in assessing the deep relationships between FOM carcinoma and the MM.

Radiologists independently collected information from all sequences acquired for both morphologic and signal alterations in the first multiparametric analysis, assigning each case to a Score category corresponding to the following features:

- Distance between the tumor and the MM > 5 mm.
- Distance < 5 mm, but not in contact.
- Tumor is in contact with the MM.
- Bulging of the MM.
- Thinning, irregularity, or non-full-thickness signal alteration of the MM.
- Full-thickness signal alteration of the MM.
- Signal alteration beyond the MM in the submandibular space.

A blinded consensus evaluation followed their multiparametric assessment, and the score allocation was correlated with the histopathological report concerning MM infiltration. Furthermore, they provided a definitive diagnosis (“positive” or “negative”) for suspected muscle infiltration. Secondly, all cases rated from 4 to 7 were re-assessed by the less experienced radiologist according to a single-sequence examination and independently re-scored to infer which among the following sequences was the most reliable: T2w, FS T2w (FS-T2), and FSCE T1w (FSCE-T1). We conducted statistical analysis on both scoring methods.

### Statistical analysis

We retrieved data from the electronic health record management program of the Local Health Administration from patients who provided a data processing agreement. After anonymization via radiological numerical code, the data was collected and saved in an encrypted file.

To correlate our MRI score rating with the histopathological findings of muscle infiltration, we considered Spearman’s rank coefficient (Rho).

The ability of our MRI score to predict MM infiltration was assessed by analyzing receiver operating characteristic (ROC) curves and comparing the area under the curves (AUC), according to DeLong. As a result, we determined the optimal cut-off to define sensitivity, specificity, and positive and negative predictive values (PPV and NPV) for our score.

The Mann–Whitney *U*-test was used to ensure that the definitive judgments of infiltration did not overlap for categories on either side of this cut-off, thus confirming the validity of the features described in the score in guiding radiologists’ infiltration verdict. We used Cohen’s *K* statistical coefficient to analyze inter-observer concordance between radiologists, considering values greater than 0.75 as excellent, moderate between 0.75 and 0.40, and poor below 0.40.

Sensitivity, specificity, PPV, and NPV were then estimated also for the sequence-selected assessment, and differentiated for the three aforementioned sequences in those cases initially scored between 4 and 7.

Statistical significance was considered for *p* values < 0.05. Statistical analysis was conducted using SPSS software (version 28.0, IBM) and SciStat (© 2021 MedCalc Software Ltd).

## Results

### Population

The original sample consisted of 81 individuals, with 28 women (34.6%) and 53 men (65.4%), resulting in a sex ratio of approximately 1:1.9, in line with epidemiological data found in the literature [17]. The overall mean age at diagnosis in this cohort was 67.4 years (standard deviation [SD], 11.4; median [Mdn], 69.0; interquartile range [IQR], 15.4). Differentiating for sex, women were on average 70.1 years (SD, 10.9; Mdn, 72.0; IQR, 9.4) and men were on average 66.0 years (SD, 11.5; Mdn, 67.3; IQR, 14.7).

After applying inclusion criteria, we obtained a sample of 52 patients. The sex distribution, with 30.8% women (*n* = 16) and 69.2% men (*n* = 36), and the overall mean age at diagnosis, which was 66.4 years (SD 11.9; Mdn 67.4; IQR 16.1), did not show significant differences from the source population (Student’s *t*-test: *p* = 0.652 and *p* = 0.476, respectively). The mean age at diagnosis changed to 70.4 years (SD 10.1; Mdn 72.9; IQR 13.0) for women and 64.7 years (SD 12.4; Mdn 65.4; IQR 16.0) for men.

### 7-Point-score

**Fig. 1:**
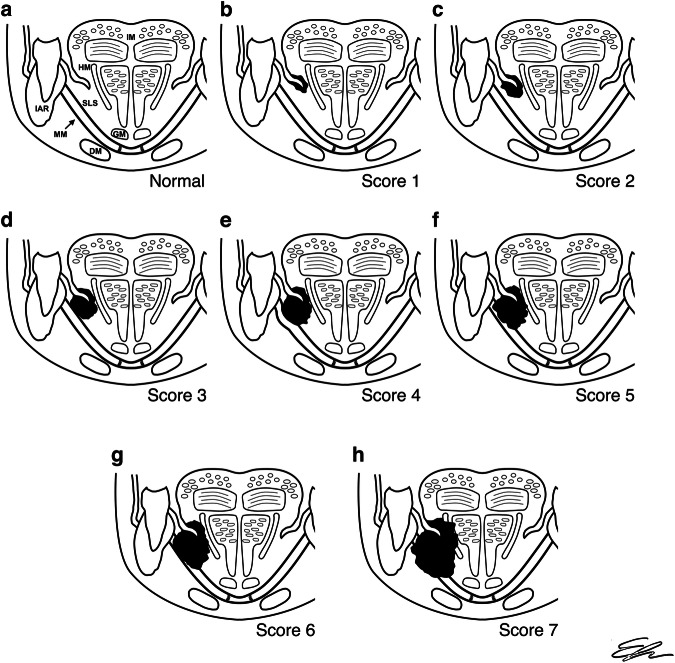
**a**–**h** In the scheme above are depicted eight images: the first on the left (**a**) is representative of normal oral cavity anatomy, with an arrow pointing to the MM, whereas the subsequent ones, **b**–**h** underline the differences between the seven categories in our score. MM, mylohyoid muscle; IAR, inferior alveolar ridge; DM, digastric muscle; GM, genioglossus muscle; HM, hyoglossus muscle; SLS, sublingual space; IM, intrinsic muscles

**Fig. 2:**
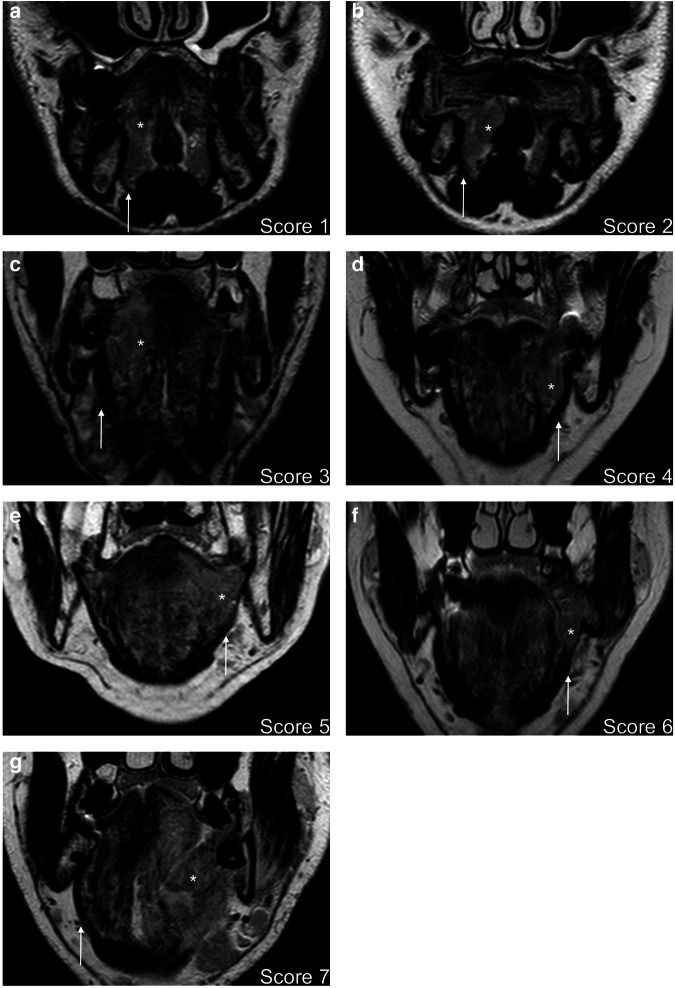
**a**–**g** We present selected T2-weighted coronal MR images from seven patients diagnosed with OSCC infiltrating the FOM. Each figure represents a specific score category in order. The tumor is identified by an asterisk in the images, while the MM’s profile is marked with an arrow (please note that in **g**, as the normal anatomy of the left FOM is subverted, the arrow points to the contralateral side of MM)

Applying our Score (Fig. 1) to the MRI sample assessment, we obtained the following distribution of cases in its seven categories: 25.0% for category-1 (*n* = 13), 5.8% for category-2 (*n* = 3), 9.6% for category-3 (*n* = 5), 9.6% for category-4 (*n* = 5), 23.1% for category-5 (*n* = 12), 13.5% for category-6 (*n* = 7), and 13.5% for category-7 (*n* = 7) (Fig. 2).

The histopathological analysis showed 21.2% of the sample (*n* = 11) exhibiting positivity for infiltration of the MM, which was distributed in the highest score categories as follows: 36.4% in category-7 (*n* = 4), 27.3% in category-6 (*n* = 3), 27.3% in category-5 (*n* = 3), and 9.1% in category-4 (*n* = 1); none tested positive in categories-1, -2 and -3 (*n* = 21). Regarding the correlation between the consensus rating and the histopathological finding, we calculated a Spearman’s Rho correlation coefficient of 0.485 (95% CI: 0.24–0.67; *p* < 0.001).

**Fig. 3:**
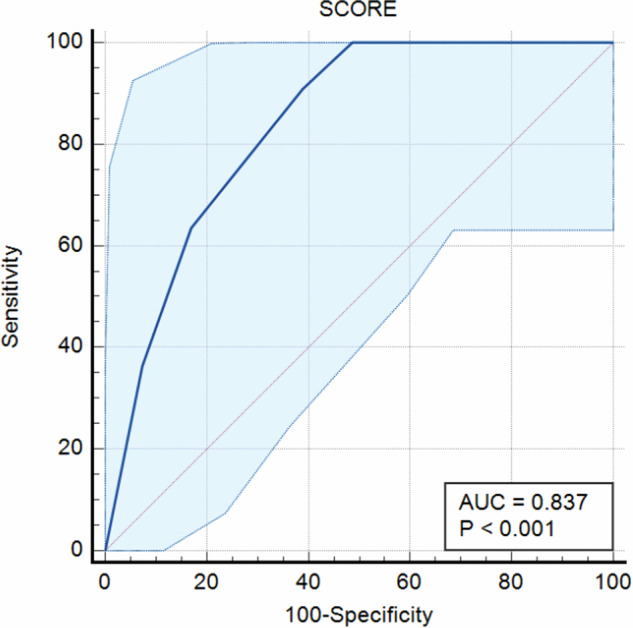
ROC for prediction of MM infiltration based on the application of the 7-point score, with the blue line portraying the best cut-off obtained (> 4). AUC, area under the curve

**Fig. 4:**
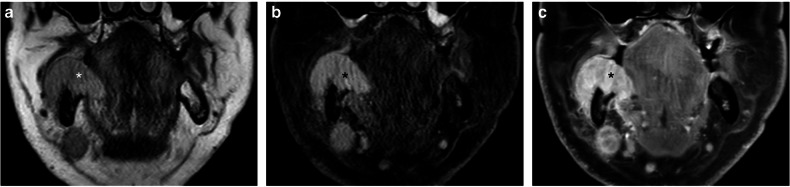
**a**–**c** Selected coronal T2w (**a**), FS-T2w (**b**), and FSCE-T1w (**c**) images of the same patient in comparable planes. A wide right inferior alveolar ridge tumor (asterisk) is seen, extending to the ipsilateral FOM, infiltrating the mandibular bone and sublingual space. The tumor front reaches the mandibular insertion of the MM, and signal alterations seem to involve its full-thickness, especially in FS-T2w (**b**) and FSCE-T1w (**c**) sequences, thus leading to a score of 7 at the multiparametric assessment. It was, therefore, a false positive radiological finding, given the absence of pathologic involvement of the muscle on histopathological examination

**Fig. 5:**
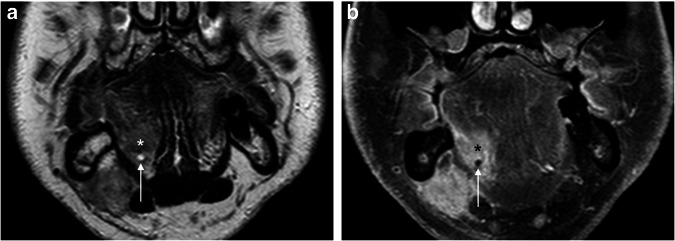
**a**, **b** Selected coronal T2w (**a**) and FSCE-T1w (**b**) images of the same patient in comparable planes. On the right, an extended anterior FOM tumor (asterisk) is seen, infiltrating the sublingual space, resulting in Wharton’s duct obstruction (arrows). Signal alterations are observed on both the sublingual and submandibular sides of the MM layer, thus leading to a score of 7. This was, therefore, a false positive radiological finding

ROC curve analysis identified a score > 4 as the best cut-off for predicting unsafe surgical margins (AUC = 0.837; CI: 0.71–0.92; standard error according to DeLong 0.0562; *p* < 0.001) (Fig. 3); with this cut-off, the score application in consensus assessment predicted MM infiltration with a sensitivity of 90.91% (CI: 0.57–0.99) and a specificity of 60.98% (CI: 0.45–0.76), diagnostic accuracy of 67.31% (CI: 0.53–0.80), a PPV of 38.46% (CI: 0.21–0.59) (Figs. 4 and 5) and a NPV of 96.15% (CI: 0.78–0.99). Table 1 compares these diagnostic values with those obtained by applying the same cut-off to the preliminary independent assessments of the two radiologists.

**Table 1:** Diagnostic values in comparison: independent radiologist 1, radiologist 2, and consensus assessment

Diagnostic results	Radiologist 1 score application	Radiologist 2 score application	Consensus score application
Sensitivity	90.91% (CI: 0.57–0.99)	81.82% (CI: 0.48–0.97)	90.91% (CI: 0.57–0.99)
Specificity	56.10% (CI: 0.40–0.71)	60.67% (CI: 0.44–0.75)	60.98% (CI: 0.45–0.76)
PPV	35.71% (CI: 0.19–0.56)	36.00% (CI: 0.19–0.57)	38.46% (CI: 0.21–0.59)
NPV	95.83% (CI: 0.77–0.99)	92.00% (CI: 0.74–0.99)	96.15% (CI: 0.78–0.99)
Diagnostic accuracy	63.46% (CI: 0.49–0.76)	65.38% (CI: 0.51–0.78)	67.31% (CI: 0.53–0.80)

*PPV* positive predictive value, *NPV* negative predictive value, *CI* confidence interval (95%)

The Mann–Whitney *U*-test rejected the hypothesis that the data on either side of this cut-off overlap (*p* < 0.00001), confirming that categories 5–7 suggest MM infiltration, while categories 1–3 suggest its integrity.

Furthermore, despite the limited sample size, there was a satisfactory inter-observer concordance, as demonstrated by a Cohen’s Kappa statistical coefficient of 0.72 (CI: 0.58–0.86). Excluding one-step discrepancies that are clinically insignificant (e.g., due to minimal differences in measurement), the result is even more encouraging, increasing to 0.83 (CI: 0.72–0.95).

We performed a single-sequence assessment to identify the most reliable one for sensitivity and specificity among 30 patients (out of 31) having a score ≥ 4 at the multiparametric evaluation. Only one case was excluded due to the suboptimal quality of some sequences. We rated their MRI again according to our Score, one at a time, using the following sequences, resulting in increased heterogeneity of true-positive distribution in categories from 4 to 7:

- T2wi: 67% in category-7; 43% in category-6; 25% in category-5; and 13% in category-4.
- FS-T2wi: 45% in category-7; 29% in category-6; and 50% in category-5.
- FSCE-T1wi: 45% in category-7; 29% in category-6; and 40% in category-5.

Among them, the T2-weighted sequence has demonstrated the highest diagnostic values, with a sensitivity of 90.91% (CI: 0.57–0.99), specificity of 70.00% (CI: 0.53–0.82), and diagnostic accuracy of 74.51% (CI: 0.60–0.86). The diagnostic values obtained for each sequence are displayed in Table 2.

**Table 2:** Diagnostic values in comparison: multiparametric consensus assessment and single-sequence assessment

Diagnostic results	Multiparametric consensus score application	Single-sequence independent radiologist 1 score application		
		T2wi	FS-T2wi	FSCE-T1wi
Sensitivity	90.91% (CI: 0.57–0.99)	90.91% (CI: 0.57–0.99)	100.00% (CI: 0.68–1)	100.00% (CI: 0.68–1)
Specificity	60.98% (CI: 0.45–0.76)	70.00% (CI: 0.53–0.82)	58.97% (CI: 0.42–0.74)	55.26% (CI: 0.38–0.71)
PPV	38.46% (CI: 0.21–0.59)	45.45% (CI: 0.25–0.67)	40.74% (CI: 0.23–0.61)	39.29% (CI: 0.22–0.59)
NPV	96.15% (CI: 0.78–0.99)	96.55% (CI: 0.80–0.99)	100.00% (CI: 0.82–1)	100.00% (CI: 0.81–1)
Diagnostic accuracy	67.31% (CI: 0.53–0.80)	74.51% (CI: 0.60–0.86)	68.00% (CI: 0.53–0.80)	65.31% (CI: 0.50–0.78)

*PPV* positive predictive value, *NPV* negative predictive value, *CI* confidence interval (95%)

### Discussion

This study examined the effectiveness of a 7-point scoring system applied to preoperative MR imaging of SCC of the FOM to characterize changes in the MM and establish a reliable predictive methodology for its infiltration. We observed a positive correlation between the major categories of our MRI Score and the histopathological evidence of infiltration of the MM. The analysis of the ROC curves indicated that a score > 4 is a robust predictor of its infiltration. Crucially, no positive cases were identified in patients with a score < 4.

In contemplating the inclination towards the infiltration of the MM within categories 5–7, juxtaposed with a proclivity towards assessing its structural integrity within categories 1–3, the decision within category 4 (bulging) becomes notably intricate. This complexity arises firstly from the restricted sample size, where the discovery of a single histopathologically positive case within this category detrimentally impacted the sensitivity and specificity values obtained in the analysis.

The achievement of an excellent NPV but low specificity, particularly in the higher categories (5–7), conveys an important message: there is a high probability that the muscle is intact in categories ≤ 4, whereas in those > 4, there is increasing suspicion of infiltration as the category increases, but with a significant chance that the muscle may not be involved on histopathological examination, even with advanced imaging.

Unlike other studies, like that of Kim et al [16], that exclusively concentrated on T2w images, our study undertook a preliminary examination of all accessible sequences to allocate relative ratings to all cases before advancing to a single-sequence assessment.

The multiparametric evaluation ensured a well-rounded analysis of the MM, particularly useful in cases with suboptimal quality of some sequences. Nevertheless, some may have adversely affected the assignment to Score categories; in particular, in the following single-sequence examination, FS-T2wi and FSCE-T1wi conditioned an upstage in 10 and 12 cases, respectively: it probably depended on signal alterations due to the presence of edema and or reactive hyperemia in peritumoral tissues. However, the separate assessment of TSE-T2w images led to the downstaging of seven cases compared to the multiparametric one, revealing a more distinct correlation between the Score and histopathological data. Besides, by cross-validating FS-T2w and FSCE-T1w images (high contrast resolution) with TSE-T2w images (high spatial resolution), it was easier to identify smaller lesions and the relationships between the tumor front and the profile of the MM.

Sometimes, DWI allows an accurate differentiation of the actual neoplastic area from the surrounding altered tissues, compared with FS-T2wi and FSCE-T1wi, by discriminating tumoral restricted diffusion. Our standard protocols include the acquisition of DWI images in the axial plane. However, the preferred plane for evaluating the MM is coronal; therefore, they were not essential in this study.

Due to a favorable ratio between spatial and contrast resolution, T2wi emerged as the key sequence in case classification, allowing excellent visualization of the MM. It also resulted in higher specificity and PPV, while NPV was comparable to multiparametric assessment, being crucial in the assignment of intermediate classes (3–5). If the tumor-determined signal alteration extends to involve the sublingual gland in contact with the MM, which is particularly exalted in FS-T2wi and FSCE-T1wi, we gave importance to the TSE-T2wi morphologic assessment, focusing both on possible thinning, better seen in the coronal plane, and on integrity and symmetry in the axial plane.

It can be challenging to determine the significance of signal changes in the FOM tissues, which can sometimes surround the MM without its overt involvement, particularly evident in cases of concurrent mandibular infiltration. This complexity might account for some false-positive cases assigned to higher categories (e.g., the 7th), where we expected a greater specificity. While the signal alterations highlighted can depend on the presence of peritumoral reactive edema/hyperemia, the morphological alterations brought about by the tumor front on the MM (thinning/irregularity) deserve separate mention. The evidence in this study leads one to ponder the possibility that this muscle is quite resistant to neoplastic spread and that, despite being compressed and altered in its morphology, it can still preserve its integrity.

It’s influential information for the clinician: even when the MRI is highly suspicious for muscle infiltration, a considerable percentage of cases may turn out unaffected on histopathological examination. MM infiltration is indeed one of the determining factors in surgical planning: if the surgeon knows that he can spare the muscle, the approach could be transoral by discontinuous resection; conversely, MM infiltration involves a full-thickness resection of the FOM by compartmental surgery (*en-bloc*), with the creation of complete communication between the oral cavity and the neck, thus the need to set up a flap to support the oral cavity structures [8].

The finding that perhaps deserves the most attention is the absence of positive cases at histopathologic analysis among those classified with a score of 1–3, including those in which the tumor was in contact with the MM; in truth, we expected some false negatives to occur. That is essential, particularly from the point of view of surgery. It is even more significant when considering that among these 21 cases, 42.9% (*n* = 9) were locally advanced carcinomas, of which 3 (14.3%) were at stage pT4a and 6 (28.6%) at stage pT3 (TNM 8th Edition). In the absence of morphological or signal changes in the MM, even when the tumor is in immediate proximity, it is still reasonable to infer the integrity of the muscle. Thus, it becomes feasible to contemplate a more conservative treatment approach despite the advanced tumor stage.

Our study has limitations. First, due to its retrospective nature, we could not determine the clinical impact of our results. Secondly, because of the small sample size, individual cases may have disproportionately impacted our results, particularly in category 4. Moreover, despite the recent availability of a higher-performance scanner (3-T) at our hospital, the retrospective review of a historical cohort of patients meant that examinations performed with a 1.5-T scanner were a significant proportion of cases (84.6%). However, although the 3-T-scanner provides higher spatial resolution [18, 19], it is also more susceptible to artifacts, like those associated with tongue motion, swallowing, or breathing [20].

A final consideration that we wondered about in this study is the frequent presence of anatomical variants and defects in the compactness of MM, known as clefts. Their potential impact on the diagnostic complexity of this anatomical region cannot be overlooked since it is complicated to determine whether they may contribute to tumor progression when ipsilateral to it. In our sample, we identified at least one cleft in 19 patients (36.5%), unilateral in 63.2% (*n* = 12), and bilateral in 36.8% (*n* = 7), for a total of 26 clefts identified, values overlapping with the literature [7, 21–24].

The anatomical complexity of this region, compounded by the presence of the sublingual gland attached to the MM and the frequent variability in muscle conformation itself, presents a considerable challenge in interpreting the relationships of the MM with FOM cancers. Due to these reasons, there was a necessity for a uniform and standardized language, as proposed in this study, in the form of a score.

In conclusion, the 7-point score system we proposed for preoperative MRI assessment shows promise in predicting surgical margins in patients affected by SCC of the FOM, with the particular benefit of T2-weighted sequences. However, its low specificity mandates careful consideration for treatment decisions despite displaying excellent NPV. With optimal interobserver agreement, our score provides consistent radiological information for surgeons.

Our study does not aim to alter the current TNM classification. However, it brings attention to clinical aspects of OSCC of particular relevance. The infiltration of deep structures, such as the MM, does not necessarily limit the surgical procedure; this could hypothetically suggest a more advanced stage, however, without prejudicing the resectability of the tumor itself. While the involvement of the MM may not be considered in staging, it may impact the surgical approach and, hence, is the focus of the scoring system we proposed.

However, in our study, we performed a retrospective radiological assessment of preoperative images, and thus we could not fully evaluate the clinical significance of our results. Further prospective studies on larger populations are warranted to refine acquisition protocols and confirm the usefulness of this method in staging and treatment planning for OSCC.

## Supplementary information

ELECTRONIC SUPPLEMENTARY MATERIAL

## Abbreviations

AUC: Area under the curve
CI: Confidence interval
CT: Computed tomography
DOI: Depth of invasion
FOM: Floor of the mouth
FS: Fat-suppressed
FSCE: Fat-suppressed contrast-enhanced
IQR: Interquartile range
Mdn: Median
MM: Mylohyoid muscle
MRI: Magnetic resonance imaging
NPV: Negative predictive value
OSCC: Oral squamous cell carcinoma
PPV: Positive predictive value
ROC: Receiver operating characteristic
SCC: Squamous cell carcinoma
SD: Standard deviation
