# Supplementary material for: MRI-based assessment of the mylohyoid muscle in oral squamous cell carcinoma, a 7-point scoring method
Source: Eur Radiol. 2024 Aug 29;35(4):2065–73. doi: 10.1007/s00330-024-11016-8 (PMC11913961; doi:10.1007/s00330-024-11016-8)
Supplement: Supplementary file 1 — ELECTRONIC SUPPLEMENTARY MATERIAL [file 330_2024_11016_MOESM1_ESM.docx]

# Supplementary Material

## ADDITIONAL FIGURES


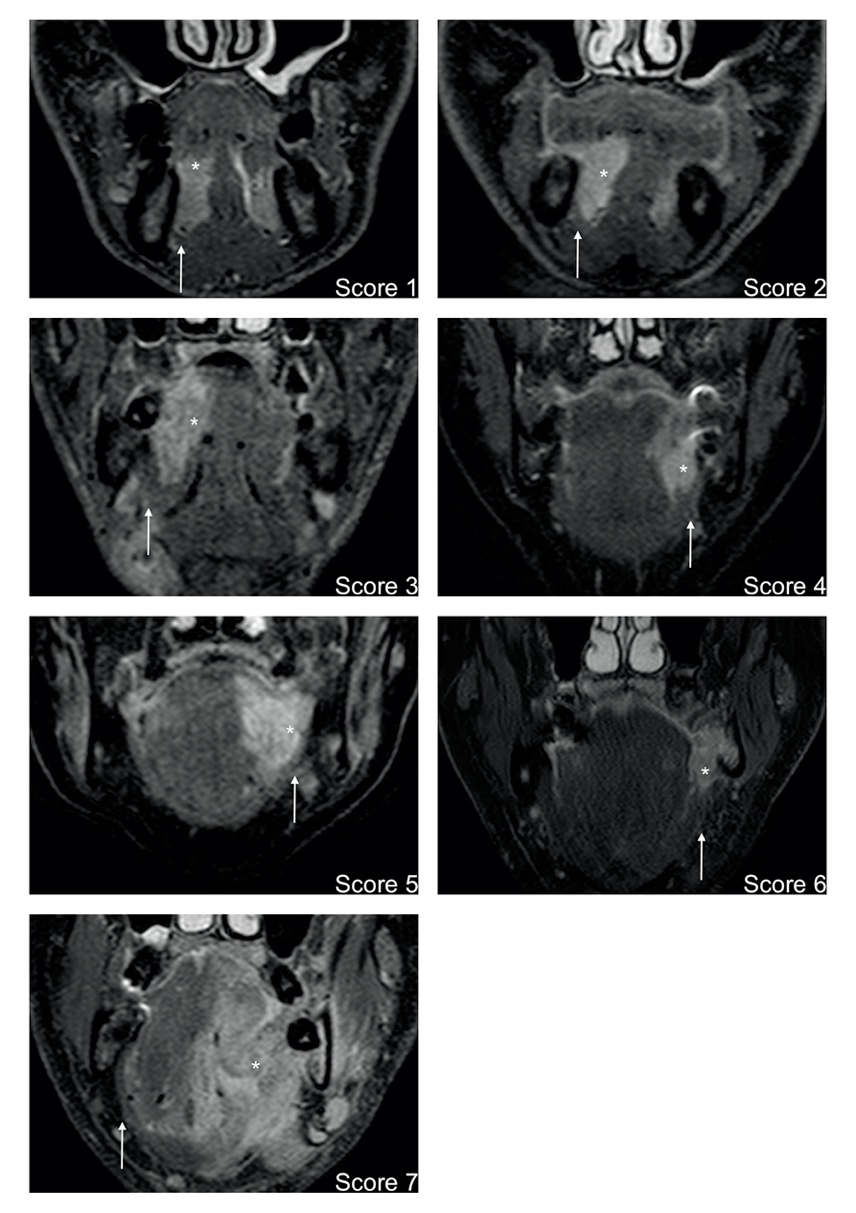


a

b

c

d

e

f

g

**Fig.S1 (S1a-S1g)**: selected FS-T2w coronal MR images of the same patients of Figure 2 and Figure S2, presented on comparable planes, with each figure representing the specific Score category. The tumor is identified by an asterisk, and the mylohyoid muscle’s profile by an arrow (exclusively in **Fig.S1g** the arrow points to the contralateral belly of MM).

**Fig.S2 (S2a-S2g)**: selected FSCE-T1w coronal MR images of the same patients of Figure 2 and Figure S1, presented on comparable planes, with each figure representing the specific Score category. The tumor is identified by an asterisk, and the mylohyoid muscle’s profile by an arrow (exclusively in **Fig.S2g** the arrow points to the contralateral belly of MM).


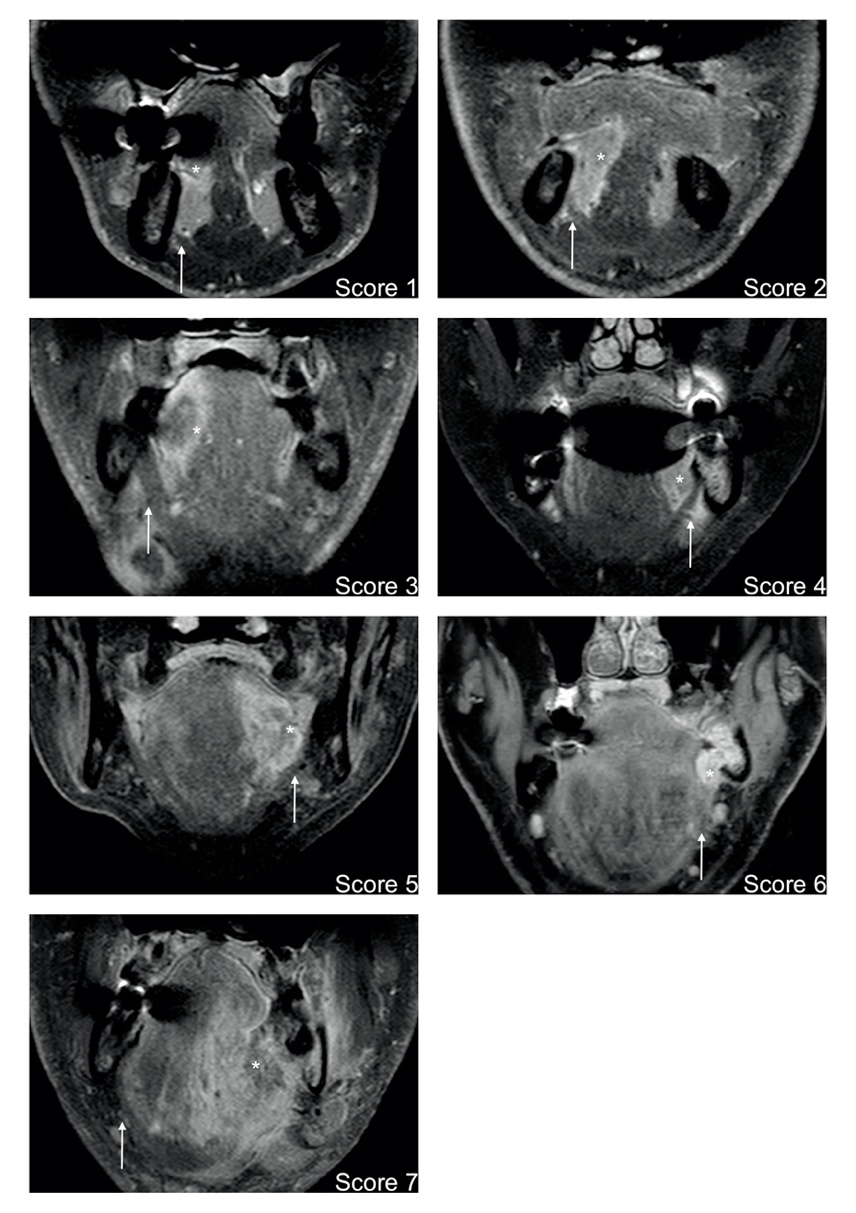


a

b

c

d

e

f

g
